# Use of *Galleria mellonella* as a Model Organism to Study *Legionella pneumophila* Infection

**DOI:** 10.3791/50964

**Published:** 2013-11-22

**Authors:** Clare R. Harding, Gunnar N. Schroeder, James W. Collins, Gad Frankel

**Affiliations:** ^1^Center for Molecular Bacteriology and Infection, Imperial College London

**Keywords:** Infection, Issue 81, Bacterial Infections, Infection, Disease Models, Animal, Bacterial Infections and Mycoses, *Galleria mellonella*, *Legionella pneumophila*, insect model, bacterial infection, Legionnaires' disease, haemocytes

## Abstract

*Legionella pneumophila*, the causative agent of a severe pneumonia named Legionnaires' disease, is an important human pathogen that infects and replicates within alveolar macrophages. Its virulence depends on the Dot/Icm type IV secretion system (T4SS), which is essential to establish a replication permissive vacuole known as the *Legionella* containing vacuole (LCV). *L. pneumophila* infection can be modeled in mice however most mouse strains are not permissive, leading to the search for novel infection models. We have recently shown that the larvae of the wax moth *Galleria mellonella *are suitable for investigation of *L. pneumophila* infection. *G. mellonella* is increasingly used as an infection model for human pathogens and a good correlation exists between virulence of several bacterial species in the insect and in mammalian models. A key component of the larvae's immune defenses are hemocytes, professional phagocytes, which take up and destroy invaders. *L. pneumophila* is able to infect, form a LCV and replicate within these cells. Here we demonstrate protocols for analyzing *L. pneumophila* virulence in the *G. mellonella* model, including how to grow infectious *L. pneumophila*, pretreat the larvae with inhibitors, infect the larvae and how to extract infected cells for quantification and immunofluorescence microscopy. We also describe how to quantify bacterial replication and fitness in competition assays. These approaches allow for the rapid screening of mutants to determine factors important in *L. pneumophila* virulence, describing a new tool to aid our understanding of this complex pathogen.

**Figure:**
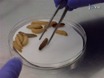


## Introduction

Animal models of infection have proved invaluable in the determination of bacterial virulence factors. However, invertebrate models have gained increased attention as a viable alternative to traditional mammalian models of infection. The larvae of the wax moth, *Galleria mellonella* is increasingly being used to study a number of important human pathogens, including Gram-positive^1^ and Gram-negative bacteria^2,3^ and several pathogenic fungi^4,5^. Using an insect model has a number of advantages over traditional mammalian models, as an invertebrate, *G. mellonella *is not subject to the ethical limitations of mammalian models. In addition, the larvae can be easily maintained, infected by injection without anesthesia, undergo pretreatment with chemical inhibitors^6^ and sustain incubation at 37 °C^7^. Interestingly, a good correlation between the pathogenicity of several microorganisms in *G. mellonella* and mammalian models of infection has been established^2,8^. The increased understanding of the immune system of *G. mellonella *has also assisted in the characterization of this model organism. Although insects do not have an adaptive immune system as found in mammals, they do have sophisticated cellular and humeral defenses including the production of antimicrobial peptides^9^. Hemocytes are the major mediator of cellular defenses and are the most numerous cell type found in the hemolymph (or blood) of *G. mellonella*^10^, These cells are professional phagocytes and perform similar functions to human macrophages and neutrophils by both taking up and degrading bacteria in a phago-lysosomal compartment^10,11^ and forming nodules around invading bacteria, physically restricting bacterial replication^12^.

*Legionella pneumophila* is a respiratory pathogen that causes severe pneumonia (Legionnaires' disease) in susceptible populations such as the elderly or immunocompromised^13^. *Legionella* is found ubiquitously in both environmental and man-made water sources, where it is a pathogen of various species of fresh water amoebae^14,15^. *Legionella* survives and replicates within these professional phagocytes by utilizing a multi-protein complex known as the Dot/Icm (defective in organelle trafficking/intracellular multiplication) type 4 secretion system (T4SS) to translocate over 275 effector proteins into the host cell^16-20^. These proteins serve to subvert the normal host cell phagocytic pathways, leading to the creation of the *Legionella* containing vacuole (LCV). The LCV avoids fusion with lysosomes and instead recruits endoplasmic reticulum (ER)-derived vesicles, resulting in a specialized compartment that resembles the rough ER^21,22^. *L. pneumophila* is considered an accidental human pathogen; the same strategies that allow it to replicate within amoebae, also allow replication in human alveolar macrophages^23^.

Mammalian hosts have been characterized as models for human *Legionella* infection including mice and guinea pigs^24,25^. However, the majority of mouse strains are resistant to *Legionella* infection^26^ with the exception of the inbred albino A/J mouse, which develops a mild, self-limiting infection^24^. Although the guinea pig model more closely resembles human disease^25^, the lack of mutants and increased cost discourages their use^27^. In addition, several invertebrate models have been developed for *Legionella pneumophila* infection including *Caenorhabditis elegans*^28^, *Drosophila melanogaster^29^* and several species of amoebae^30-32^. However, these models have weaknesses, virulence in the *C. elegans* system is not Dot/Icm-dependent^28^, limiting the utility of this model. The *Drosophila* model has proved effective in investigating bacterial virulence factors^29^ and appears to be promising however, this model has not been fully characterized. Single celled amoebae are the environmental hosts of *L. pneumophila* and are ideal for investigating the action of virulence factors at a molecular level^33^ however lack several important mediators of the mammalian host cell response to infection such as caspases^34^. The weaknesses of the existing models, along with the high cost and ethical concerns related to mammalian experimentation, has led to the search for other appropriate model organisms^29,35^.

We have recently demonstrated that *G. mellonella* is a suitable model for *L. pneumophila* pathogenesis^36,37^. This protocol details the experimental techniques used for infecting *G. mellonella* larvae, analyzing larval morality, extracting hemocytes for counting and immunofluorescence and determining replication by viable CFU counts from infected larvae.

## Protocol

### 1. Preparation of *L. pneumophila* for Infection

1. Prepare charcoal yeast extract (CYE) plates (2 g/L activated charcoal, 10 g/L yeast extract, 13 g/L agar, 10 g/L N-(2-acetamido)-2-aminothanesulfonic acid (ACES), 1 g/L α-ketoglutarate, 0.4 g/L L-cysteine HCl and 0.25 g/L ferric pyrophosphate, pH 6.9). Note: If required, add kanamycin (25 μg/ml) and/or chloramphenicol (6 μg/ml) to CYE plates.
  1. Carry out all *L. pneumophila* work at biosafety containment level 2 (BSL-2) in a microbial safety cabinet (MSC) in compliance with local rules.
2. Streak *L. pneumophila* from -80 °C glycerol stocks onto CYE plates
3. Incubate plates for 4 days at 37 °C. Note: Incubation of plates for 4 days significantly increases the virulence of *L. pneumophila* in *G. mellonella *over incubation for 3 days.
4. One day before infection, resuspend one loop full of bacteria (containing several colonies) in 1 ml of prewarmed (37 °C) ACES yeast extract (AYE) broth and measure the absorbance at 600 nm (OD_600_) using a spectrophotometer.
5. Inoculate a fresh 3 ml AYE culture (with antibiotics if required) to a final OD_600_ of 0.1.
  1. Include a media only control to ensure sterility of the media.
6. Incubate at 37 °C in a shaking incubator at 200 rpm for 21 hr. Note: Bacteria should be grown to post-exponential phase for infection^38^. Growing for 21 hr allows experiments to be standardized. Note: If required for protein induction, add 0.5 mM isopropyl β-D-1-thiogalactopyranoside (IPTG) overnight.
7. Measure the OD_600_ (bacteria should be in post-exponential growth phase, OD_600_ 2.5-3).
8. Dilute bacterial culture to give 1 x 10^9^ CFU/ml in sterile Dulbecco's phosphate buffered saline (D-PBS). Note: Based on previous results, an OD_600 _of 1 corresponds to 1 x 10^9^ CFU/ml, however this should be confirmed for different *L. pneumophila* strains. Note: if induction of a protein from a plasmid is required, add 1 mM of IPTG to the inoculum.
9. Plate the inoculum as described in section 9 as a control to ensure the expected CFU is present in the inoculum.

### 2. Preparation of Larvae

1. Purchase sufficient *G. mellonella* larvae from a commercial supplier. Larvae are shipped at 5^th^ or 6^th^ instar stage (approximately between 2-3 cm in length) and are suitable for use immediately. Note: A method describing how to rear larvae has been described previously^39^ . Note: Larvae can be stored at room temperature for up to two weeks and do not require food. Immediately discard any larvae showing signs of pupation.
2. Prepare the container for larva by placing a circle of 10 cm filter paper in the bottom of a 10 cm Petri dish.
3. Using blunt tipped tweezers, place ten healthy larvae of approximately similar size into the Petri dish. Note: Discard unhealthy brown colored or blotchy looking insects. Healthy larvae are uniformly creamy colored with no areas of dark discoloration and are able to right themselves quickly if turned over.

### 3. Infection of *G. mellonella* Larvae

1. Prepare the injection platform by taping a circle of filter paper to the surface.
2. Securely tape a P1000 tip horizontally to the filter paper to create an injection platform. This does not need to be sterile.
3. Sterilize a 20 μl microtiter syringe by aspirating 70% ethanol and incubating for at least 10 min.
  1. Wear puncture proof gloves while injecting,
4. Remove any residual ethanol by aspirating and expelling sterile water several times.
5. Using the syringe, aspirate 10 μl of the *L. pneumophila* 1 x 10^9^ CFU/ml suspension.
6. Take one larva and gently but firmly turn it on its back, bent over the P1000 tip.
7. Place the tip of the syringe over the front, right proleg of the larvae.
8. Gently, insert the tip of the needle into the proleg, making sure that it is inside the larvae, and smoothly inject all of the syringe contents. Note: If the syringe is inserted correctly, it should be possible to pick up the larvae using only the syringe to place it into the chamber.
  1. After injection, observe the larvae for a few seconds. The larvae will start to crawl after a couple of seconds but should not excrete fluid.
9. Briefly observe the larvae a few hours post infection; infection with 10^7^ CFU *L. pneumophila* 130b does not cause any symptoms within the first 5-8 hr p.i. Therefore if larvae are turning grey/black before this point, the experiment should be discontinued. Note: Inoculation of larvae with 1 mM IPTG does not affect larval viability over the course of the experiment.
10. In the same manner, inject a total of 10 larvae per condition including 10 larvae injected with D-PBS to serve as a control to analyze larval mortality.
11. Tape Petri dishes closed and place in secondary containment.
12. Incubate in a standard bacterial incubator at 37 °C, for the duration of the experiment.

### 4. Pretreatment of Larvae with a Chemical Inhibitor

1. Prior to infection, prepare larvae for injection as described in section 3.1-3.6.
2. Inject 10 μl of a 100 μM solution of Cytochalasin D into the front, left proleg of 10 larvae. Note: Inhibitor is injected into a different proleg from bacterial suspension to reduce injury to the larvae.
3. Inject 10 μl of DMSO into 10 larvae as a control.
4. Incubate larvae at 37 °C for 4 hr.
5. Inject pretreated larvae as described in section 3 into the front, right proleg with either 1 x 10^7^ CFU of WT *L. pneumophila* or a PBS control.

### 5. Analysis of Larval Mortality

1. At 18 hr post infection (p.i.), examine all infected larvae for mortality.
2. To check mortality, use blunt tipped tweezers to turn over the larvae and look for movement of the legs, healthy larvae should right themselves quickly. Pigmentation indicates a strong immune reaction to infection. If there is any movement, count as alive.
3. Record number of dead and alive larvae.
4. Repeat this process at all other time points chosen. Note: If incubation is continued for more than three days, pupae may be seen. Remove any pupae and euthanize by freezing at - 20 °C before metamorphosis can occur.

### 6. Extraction of Hemolymph

1. Randomly select three larvae and place into a 14 ml tube at selected time points such as 5 and 18 hr p.i.,
2. Place this tube on ice for 5-10 min, until no movement of the legs of the larvae can be observed.
3. Place the anesthetized larvae onto a Petri dish and, using a scalpel, make an incision between two segments near the tail of the larvae.
4. Squeeze the larvae into a sterile 1.5 ml centrifuge tube to collect the hemolymph.
  1. Pool hemolymph from at least three individuals. One larvae gives between 15-50 μl of hemolymph depending on size. Note: During hemolymph extraction it is very easy to disrupt the gut, resulting in potential contamination of the samples. Reduce contamination by cutting the larvae near the tail (away from the gut) however, antibiotic selection will always be required when plating out the bacteria. Note: To prevent hemolymph from turning brown and coagulating, process the hemolymph within 10 min after collection.
5. Discard the larval body into a new 14 ml Falcon tube, seal and place at - 20 °C overnight to ensure the larvae are dead.
6. Autoclave dead larvae and dispose according to local rules.

### 7. Determination of Hemocyte Viability

1. Extract hemolymph as described above.
2. Mix 20 μl of extracted hemolymph with 20 μl of 0.02% (v/v) Trypan blue in PBS in one well of a 96 well plate.
3. Incubate for 5 min at RT.
4. Load 10 μl of hemolymph onto a hemocytometer and count viable (not blue) cells.
5. Count each sample in triplicate to reduce error.

### 8. Processing of Extracted Hemocytes for Immunofluorescence Microscopy

1. Mix the pooled hemolymph extracted from at least three larvae and pipette onto a 10-15 mm glass coverslip in a 24-well plate. Note: Coverslips do not require treatment as hemocytes can adhere to glass .
2. Add 0.5 ml of D-PBS and mix well by pipetting up and down.
3. Centrifuge the plate for 10 min at 500 x g at room temperature (RT) using an aerosol-tight centrifuge plate holder.
4. Examine each well using an inverted microscope to check that the hemocytes have adhered.
5. Remove the supernatant and carefully wash the cells three times by adding 0.5 ml D-PBS to the wall of the well, rocking the plate 2-3x and removing the D-PBS with a pipette.
6. Fix the cells by addition of 0.5 ml of 4% (v/v) paraformaldehyde (PFA) in PBS.
7. Incubate the cells for between 20-30 min at RT.
8. Wash the cells three times with D-PBS, as before.
9. Add 0.5 ml 15 mM NH_4_Cl in PBS to quench residual PFA and incubate at RT for 15 min.
10. Wash the cells three times with D-PBS. Note: At this stage, the coverslips can be stored overnight at 4 °C.
11. Add 0.5 ml 0.1% Triton X-100 in PBS and incubate for 5 min at RT to permeabilize cells.
12. Block for 1 hr with blocking solution (2% (w/v) BSA in PBS).
13. Incubate for 1 hr at RT in the dark with the primary antibody diluted in blocking solution at the dilution specified by the manufacturer.
14. Wash 3x with PBS.
15. Incubate for 1 hr with the secondary antibody and DAPI for visualization of bacteria as above.
16. Wash 3x with PBS.
17. Mount the coverslips using one drop of mounting reagent onto glass slides.
18. Incubate overnight in the dark at RT to fully dry the mounting solution.
19. Image slides on a fluorescence microscope.

### 9. Quantification of Bacterial CFU

1. Add 100 μg/ml spectinomycin to CYE plates to avoid contamination by gut flora. *L. pneumophila* strain 130b is naturally resistant to spectinomycin^40^.
2. Before hemolymph extraction, weigh 1.5 ml centrifuge tubes.
3. Extract hemolymph as described in section 6 and place in weighed tubes, add 1 μl of 5 mg/ml digitonin, mix well and incubate for 5 min at RT to lyse hemocytes.
4. Reweigh the tube with hemolymph and determine the weight of hemolymph extracted.
5. Perform ten fold serial dilutions of the hemolymph in sterile AYE media.
6. Using a pen, divide the base of a CYE plate into six equal sectors and label.
7. Plate three drops of 25 μl of each dilution (starting with the most dilute) in each section of the plate.
8. Incubate the plates with the lids upmost overnight at 37 °C.
9. Once the drops have dried fully, turn the plate over and incubate at 37 °C for at least a further two days.
10. Quantify the bacteria extracted by counting the colonies at each dilution and normalize to the weight of hemolymph extracted.

### 10. Determination of the Competitive Index (CI)

1. Confirm that both strains grow equally well in broth culture and on CYE agar plates prior to attempting the competitive index.
2. Prepare WT or kanamycin resistant mutant bacterial suspensions as described in section 1 and mix in a 1:1 ratio.
  1. Plate serial dilutions of the inoculum onto CYE spectinomycin (100 μg/ml) and CYE spectinomycin /kanamycin
3. Infect larvae and extract hemolymph at suitable time points as described above.
4. Determine viable counts by extracting hemolymph and plating serial dilutions onto CYE spectinomycin and CYE spectinomycin/kanamycin.
5. Calculate the competitive index (CI) as follows: CI = (mutant output/WT output)/(mutant inoculum/WT inoculum).

## Representative Results

Here it is demonstrated that *G. mellonella* is an appropriate, easy to use model to study *L. pneumophila* infection. Previously it has been shown that *L. pneumophila* virulence in macrophages, amoebae and mammalian models is dependent on the presence of the Dot/Icm secretion system ^41-43^. *G. mellonella* larvae were infected as described above and the virulence of the wild type (WT) and a Dot/Icm-deficient strain compared. Infection with 10^7^ CFU of *L. pneumophila* strain 130b resulted in 100% mortality within 24 hr post infection (p.i.). However, the *L. pneumophila* Δ*dotA* strain, which does not have a functional Dot/Icm T4SS secretion system, was avirulent in this assay (**Figure 1**). This demonstrates that *L. pneumophila* virulence in *G. mellonella* depends on the translocation of Dot/Icm effectors, making this model suitable for characterization of the function of these proteins.

Recently, it was shown that inhibition of phagocytosis by cytochalasin treatment increased the susceptibly of the larvae to infection by the yeast *Candida albicans*^6^. As *L. pneumophila* is an intracellular pathogen, it was decided to determine if uptake of the bacteria is crucial in its pathogenesis in this model. Larvae were pretreated with 10 μl of 100 μM Cytochalasin D (CyD) for 4 hr at 37 °C, then infected with 10^7^ CFU of WT *L. pneumophila *130b and mortality monitored at 24 hr p.i. Treatment with the inhibitor alone did not affect larval survival. However, pretreated, infected larvae displayed significantly greater survival (*P* = 0.0066, unpaired T-test) compared to DMSO-treated, infected insects (**Figure 2**). The effect of CyD treatment was abolished by 48 hr p.i. (results not shown); this may be due to the half-life of the drug in *G. mellonella*. This demonstrates that uptake of* L. pneumophila *into *G. mellonella* hemocytes is a crucial aspect of bacterial virulence.

In order to validate expression and determine the subcellular localization of an effector protein in *G. mellonella,* hemocytes were extracted and processed for immunofluorescence microscopy. Larvae were infected with WT and Δ*dotA L. pneumophila* 130b expressing a fragment of the well-defined T4SS effector, SidC_41-918_, fused to 4 N-terminal HA tags. This effector was demonstrated to bind the LCV via a phosphoinositide-4-phosphate-binding domain^44^. Using anti-HA (red) and anti-*Legionella* (green) antibodies, 4HA-SidC_41-918_ localized to the LCV in infected hemocytes (**Figure 3**). This localization has previously been shown in the amoebae *Dictyostelium discoideum *and in mammalian macrophages^44,45^ confirming the comparability of this model.

The importance of proteins for virulence is usually determined by comparing the growth kinetics of wild type and mutant bacteria. In order to follow the bacterial replication kinetics over the course of the infection, three larvae were sacrificed at each time point (0, 5, 18, and 24 hr p.i.), the hemolymph collected and pooled and the CFU/0.1g of extracted hemolymph determined. After an initial dip at 5 hr p.i., the CFU of the WT bacteria increases up to 24 hr p.i. however, the Δ*dotA* strain undergoes no replication and is cleared at 18 hr p.i. (**Figure 4**).

The ability of *L. pneumophila* to cause lysis of macrophages in a T4SS-dependent manner has long been documented^46^, however no similar studies have been performed *in vivo*. The concentration of circulating hemocytes was determined at 5, 18, and 24 hr p.i. Larvae were infected with WT or Δ*dotA L. pneumophila* 130b, hemocytes extracted from infected insects and viable cells counted using the trypan blue exclusion method. At 5 hr p.i. no difference in hemocyte counts between the strains could be seen (**Figure 5**). However, at 18 hr p.i. there was a significant drop in hemocyte concentration in WT, but not Δ*dotA*, infected larvae. This difference persisted at 24 hr p.i. The drop in hemocyte number, combined with the presence of intracellular bacteria as seen by immunofluorescence, suggests that *L. pneumophila* replicates within hemocytes then lyses them, allowing the bacteria to undergo several rounds of replication.


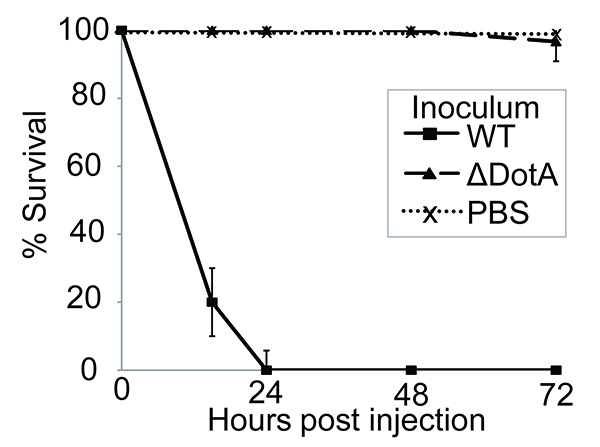
**Figure 1. Infection with *L. pneumophila* induces Dot/Icm-dependent larval mortality.** 10 larvae were infected with PBS alone or 10^7^ CFU of wild type (WT) or Δ*dotA L. pneumophila* 130b, incubated at 37 °C for 72 hr and the time of death of the larvae recorded. All larvae infected with the WT succumbed to infection within 24 hr post infection (p.i.), however no mortality was seen in larvae inoculated with PBS alone or the Δ*dotA* strain. Results are the mean of three separate experiments, ± standard deviation.


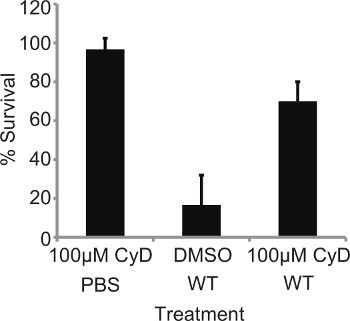
**Figure 2. Mortality is dependent on bacterial internalization.** 10 *L. pneumophila* larvae were pretreated with 10 μl of 100 μM Cytochalasin D (CyD) for 4 hr at 37 °C then infected with 10^7 ^WT and mortality monitored at 24 hr p.i. Pretreated larvae demonstrated significantly (*P* = 0.0066, unpaired T-test) reduced mortality. Results represent the mean of at four independent experiments ± standard deviations with 10 larvae per condition.


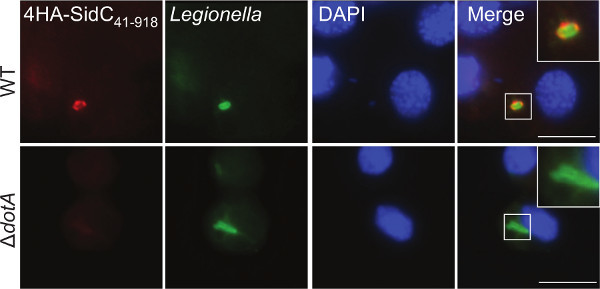
**Figure 3. Immunofluorescence imaging of effector proteins in extracted hemocytes.** Hemocytes were extracted from larvae infected with *L. pneumophila* 130b WT or Δ*dotA* expressing 4HA-SidC_41-918 _at 5 hr p.i. Cells were stained using anti-HA (red) and anti-*Legionella* (green) antibodies and DAPI DNA stain (blue) to visualize the nuclei. 4HA-SidC_41-918_ was observed surrounding WT, but not Δ*dotA*, bacteria. Scale bar 5 μm.


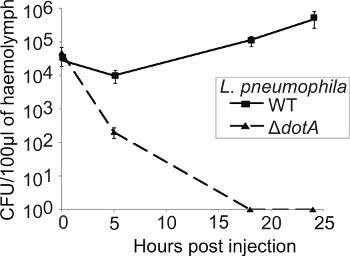
**Figure 4. *L. pneumophila* replicates within *G. mellonella* in a Dot/Icm-dependent manner. **Larvae were infected with WT or Δ*dotA L. pneumophila* and at 0, 5, 18, and 24 hr p.i. the hemolymph from three infected insects pooled, plated onto CYE plates and the CFU determined and normalized to the inoculum and to the weight of hemolymph extracted. WT *L. pneumophila* replicated over the course of the experiment while the Δ*dotA* strain was cleared within 18 hr p.i. Results are the mean of three separate experiments ± standard deviation.


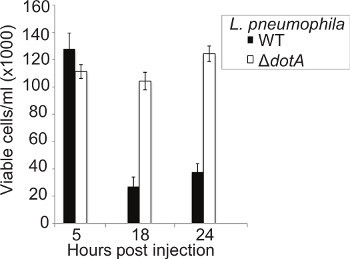
**Figure 5. Infection with WT *L. pneumophila* results in significant hemocyte destruction.** Hemocytes were extracted at 5, 18, and 24 hr p.i. from larvae infected with WT or Δ*dotA L. pneumophila* and viable cells counted using a hemocytometer. No difference in the number of cells was seen at 5 hr p.i. between the strains however at 18 hr p.i. only approximately 15% of hemocytes remain in larvae infected with the WT strain compared to the Δ*dotA* strain. Results are the mean of three separate experiments, ± standard deviation.

## Discussion

The *Galleria mellonella* larval model for *Legionella pneumophila* infection is a useful tool for *in vivo* studies of pathogenesis. Here it is shown that a number of aspects of macrophage infection can be recapitulated in the *G. mellonella* model, including the role of the Dot/Icm T4BSS in virulence and bacterial replication and the localization of the Dot/Icm-effector SidC. Additionally, we demonstrate that a chemical inhibitor of actin polymerization significantly reduces larval mortality, mimicking results obtained in macophages^47^ and supporting evidence that internalization of the bacteria is required to cause larval mortality. Previously, it has been demonstrated that the variations in virulence between *L. pneumophila* strains seen in other infection models can be verified in *G. mellonella *and that induction of virulence factors in post-exponential growth phase is required for bacterial virulence^36^, confirming that *G. mellonella *is a suitable model for *L. pneumophila* infection.

Determining the CFU of *L. pneumophila* from larvae infected either singly or in mixed infections greatly increases the utility of the model. Previously, several factors have been discovered that have subtle effects on bacterial replication in one or more models of infection^29,48-51^. Although the larvae do not possess an adaptive immune system, the presence of the innate immune response provides stronger selection compared to macrophages alone, which may serve to amplify subtle phenotypes. Therefore, it is possible that, while these strains will probably not significantly affect larval mortality, they may demonstrate decreased bacterial replication or fitness in the *G. mellonella* model. As well as replication of *L. pneumophila* in the larvae, we have shown significant hemocyte depletion late in infection. As *L. pneumophila* is expected to lyse host cells at the end of its replication cycle, measuring hemocyte depletion may also serve as an indirect measurement of bacterial replication. Hemocyte depletion has previously been correlated with insect mortality in infection^3,52^, although recent results suggest that this picture is more complex than first belived^37^. Recently, it has been shown that starvation of larvae leads to increased susceptibility to infection through a suppression of immune responses^53^. In the assays described here, larvae were not fed for the duration of the study and it is not known how well fed larvae would respond to *L. pneumophila* infection.

One of the advantages of *G. mellonella* as a model organism is the ease of extraction and quantification of hemocytes from infected larvae. Previous videos have shown various methods for extracting hemocytes from insects^54,55^ however, the method presented here is simple and suitable for immediate processing. Once extracted, hemocytes can be easily quantified, used for immunofluorescence, transmission electron microscopy^36^ or flow cytometry^56^ or cultured and infected *ex vivo*^3^ allowing the response of the cells to infection to be investigated in detail. This significantly increases the flexibility of the model. One caveat to immunofluorescence in *G. mellonella* is the limited supply of antibodies validated against *G. mellonella *proteins. However, studies have demonstrated the creation of antibodies against larval proteins^57^ and antibodies against human immune related proteins were found to recognize *G. mellonella *proteins^11^ demonstrating the potential for immunofluorescence on *G. mellonella* hemocytes.

The ease of *G. mellonella* infection allows for rapid, medium throughput screens that could be used to compare the virulence of various *Legionella* species and strains and could be used to further analyze previously identified virulence factors such as adhesion molecules^58^ or the type 2 secretion system^59^ which are required for virulence in other models. In addition, use of this model will allow the identification and further characterization of novel virulence factors including secreted and translocated effector proteins. Recently, it has been shown that phospholipase C activity of *L. pneumophila* has a role in *G. mellonella *virulence^60^ and that the Dot/Icm effector protein SdhA is required for virulence^37^. In addition, we have recently demonstrated that there is a correlation between the phenotypes observed in *G. mellonella* and in the A/J mouse strain^37^.

This underlines the value of this tool to complement environmental protozoan and unicellular host and murine infection models. The *G. mellonella *model will become even more valuable in the future, once the larval genomic sequence will be available and more genetic tools are established. Steps in this direction include the recent publication detailing the immune-related transcriptome^61^ and the formation of an initiative to advance gene silencing in *Lepidoptera* spp^62^.

By using *G. mellonella* larvae, we have a number of simple, rapid readouts of bacterial virulence that can be used to investigate the pathogenesis of *L. pneumophila*. Establishment of these assays and wider screening of *L. pneumophila* strains and serogroups will increase the utility of this new tool and will contribute to our understanding of *L. pneumophila* pathogenesis.

## Disclosures

The authors have nothing to disclose.
